# Resveratrol inhibits nonalcoholic fatty liver disease in rats

**DOI:** 10.1186/1471-230X-8-40

**Published:** 2008-09-09

**Authors:** Luis Bujanda, Elizabeth Hijona, Mikel Larzabal, Marta Beraza, Pablo Aldazabal, Nerea García-Urkia, Cristina Sarasqueta, Angel Cosme, Belen Irastorza, Alberto González, Juan I Arenas

**Affiliations:** 1 Department of Gastroenterology, University of Country Basque, Donostia Hospital, Centro de Investigación Biomédica en Enfermedades Hepáticas y Digestivas (CIBERehd), San Sebastián, Spain; 2 Department of Pathology, Donostia Hospital, San Sebastián, Spain; 3 Department of Experimental Surgery, Donostia Hospital, San Sebastián, Spain; 4 Department of Epidemiology, Donostia Hospital, San Sebastián, Spain; 5 Department of Pharmacology, Donostia Hospital, San Sebastián, Spain; 6 Department of Microbiology, Donostia Hospital, San Sebastián, Spain

## Abstract

**Background:**

The prevalence of nonalcoholic fatty liver disease (NAFLD) is high. NAFLD is linked to obesity, diabetes mellitus, and hypertriglyceridemia. Approximately 20% of patients with NAFLD will eventually develop cirrhosis. Our purpose was to investigate whether resveratrol decreased hepatic steatosis in an animal model of steatosis, and whether this therapeutic approach resulted in a decrease in tumor necrosis factor α (TNF-α) production, lipid peroxidation and oxidative stress.

**Methods:**

Male Wistar CRL: Wi (Han) (225 g) rats were randomized into three groups. A control group (n = 12) was given free access to regular dry rat chow for 4 weeks. The steatosis (n = 12) and resveratrol (n = 12) groups were given free access to feed (a high carbohydrate-fat free modified diet) and water 4 days per week, and fasted for the remaining 3 days for 4 weeks. Rats in the resveratrol group were given resveratrol 10 mg daily by the oral route. All rats were killed at 4 weeks and assessed for fatty infiltration and bacterial translocation. Levels of TNF-α in serum, hepatic malondialdehyde (MDA), oxidative stress (superoxide dismutase, glutathione peroxidase, catalase and nitric oxide synthase) and biochemical parameters were measured.

**Results:**

Fat deposition was decreased in the resveratrol group as compared to the steatosis group (Grade 1 vs Grade 3, P < 0.05). TNF-α and MDA levels were significantly increased in the steatosis group (TNF-α; 33.4 ± 5.2 vs 26.24 ± 3.47 pg/ml and MDA; 9.08 ± 0.8 vs 3.17 ± 1.45 μM respectively, *P *< 0.05). This was accompanied by increased superoxide dismutase, glutathione peroxidase and catalase and decreased nitric oxide synthase in the liver of resveratrol group significantly (*P *< 0.05 vs steatosis group). Bacterial translocation was not found in any of the groups. Glucose levels were decreased in the group of rats given resveratrol (*P *< 0.05).

**Conclusion:**

Resveratrol decreased NAFLD severity in rats. This effect was mediated, at least in part, by TNF-α inhibition and antioxidant activities.

## Background

Nonalcoholic fatty liver disease (NAFLD) is characterized by histological changes similar to those seen in subjects with alcoholic hepatitis but in whom alcohol intake is absent or poorly significant. Factors promoting NAFLD development include obesity and diabetes. From 69% to 100% of patients with NAFLD are obese [1]. NAFLD prevalence in severe obesity is greater than 30% [2,3]. Because of all these reasons, it is important to have animal models that allow us to study the effect of different substances on NAFLD.

Polyphenols have a variety of biological functions, including antioxidant, anti-inflammatory, and anticancer effects [4]. Resveratrol is a phytoalexin polyphenolic compound occurring in various plants, including grapes, berries, and peanuts. Multiple lines of compelling evidence suggest its beneficial effects on neurological, hepatic, and cardiovascular systems. The potential mechanisms responsible for its biological activities include downregulation of the inflammatory response through inhibition of the synthesis and release of pro-inflammatory mediators, modification of eicosanoid synthesis, inhibition of Kupffer cells and adhesion molecules, inhibition of inducible nitric oxide synthase and cyclooxygenase-2 (COX-2) via its inhibitory effects on nuclear factor (kappa)B (NF-kB) or the activator protein-1 (AP-1) (5,6). In our previous studies, resveratrol was seen to decrease the liver lesions and transaminase elevations caused by alcohol in mice [7].

Our purpose now was to investigate first whether resveratrol decreased hepatic steatosis in an animal model of steatosis, and second, whether this therapeutic approach resulted in a decrease in tumor necrosis factor α (TNF-α) production and oxidative stress.

## Methods

### Animals and diets

Wistar CRL: Wi (Han) male rats (Charles River) weighing approximately 225 g were used. All experiments were conducted in accordance with the Guide for the Care and Use of Laboratory Animals published by the US Public Health Service. Animals were kept on a regular 12-hour light period at a controlled temperature (25 ± 2°C). The modified diet (high carbohydrate-fat free) consisted of carbohydrates (80%, as starch), protein (16%, as casein), and vitamins and minerals (4%)(PANLAB, Barcelona, Spain). The standard diet consisted of a balanced diet containing carbohydrates (51%), protein (16%), vitamins and minerals (4%), and lipids (3%). The standard diet contained 2.9 kcal/g, and the modified diet 3.58 kcal/g. The model was based on the model reported by Delzenne in 1997 [8].

### Experimental procedures

**Figure 1:**
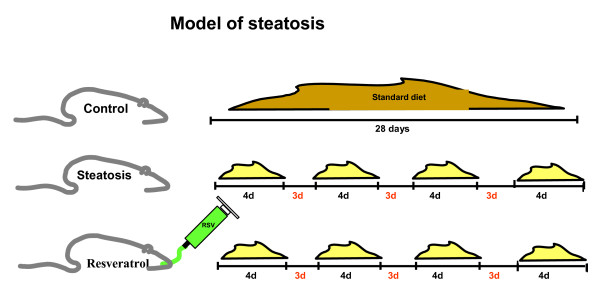
NAFLD model used in the study.

Rats were distributed into three groups: control, steatosis and resveratrol. The control group was given free access to feed and drink. The control group was fed a standard diet. The steatosis and resveratrol groups were given free access to feed and water 4 days per week, and were fasted for the remaining 3 days (access to water was only allowed). Feed consisted of a modified diet. During the dietary restriction cycles, steatosis and resveratrol groups were fed a modified diet (Figure 1). Rats in the resveratrol group were given resveratrol 10 mg daily by the oral route through an orogastric catheter. Resveratrol was diluted in 1 mL of water. Resveratrol was obtained from SIGMA Chemical, (Pool, Dorset, UK).

All rats were killed after completing 4 cycles of feeding and fasting, i.e. 28 days after study start. The timing of killing was decided based on previous trials in which hepatic damage was seen to be very high after four weeks in the steatosis group. Feed taken by the rats and weight of the animals were controlled at the end of the study. At the end of the study, all rats were anesthetized using an intraperitoneal injection of an overdose (45 mg/kg) of sodium pentobarbital.

### Pathological evaluation

A histological study was performed following a midline laparotomy to remove the liver. Liver was weighed, and liver tissue samples taken at the time the rat was killed were immediately placed in 10% buffered formalin and subsequently embedded in paraffin. Hepatic index was obtained by dividing liver weight by rat weight × 100. Liver sections were stained with hematoxylin and eosin using standard techniques. Sections were viewed without knowing the treatment group to which each animal belonged. Biopsies were classified into four grades depending on fat accumulation using the Brunt et al [9] classification, assigning grade 0 when no fat was found in the liver, grade 1 when fat vacuoles were seen in less than 33% of hepatocytes, grade 2 when 33%–66% of hepatocytes were affected by fat vacuoles, and grade 3 when fat vacuoles where found in more than 66% of hepatocytes. The deposit of fat was classified in macrovesicular, microvesicular and mixed. Two experienced pathologists blinded to the experiment evaluated all samples. Agreement between both pathologists was determined.

### Biochemical measurements

Laboratory parameters such as ALT, glucose, and albumin were measured using an automatic analyzer (Roche/Hitachi Modular Analytics, Roche Diagnostics, Mannheim, Germany) at 37°C.

Serum TNF-α levels was measured using ELISA kits (R&D Systems, Boston, MA-Catalog Number RTA00). TNF-α levels was expressed in pg/mL.

### Lipid peroxidation

Malondialdehyde (MDA) was measurement in hepatic tissue. For hepatic malondialdehyde (MDA) determination, weigh 25 mg of tissue and add 250 μl of RIPA buffer with protease inhibitors. Sonicate for 15 seconds at 40 V over ice and centrifuge at 1.600 × g for 10 minutes at 4°C. We use the supernatant for analysis. MDA was quantified using the thiobarbituric acid reaction as described by Ohkawa [10]. MDA levels were measured using Cayman's TBARS Assay Kit. MDA levels were expressed in μM.

### Oxidative stress

Superoxide dismutase, catalase, glutathione peroxidase, and nitric oxide synthase levels as oxidant/antioxidant biochemical parameters were measurement in hepatic tissue. Levels were quantified using Cayman's (Cayman Chemical Company, Ann Arbor – Michigan, USA) assay kit following the instructions of the manufacter.

### Bacterial translocation determination

Samples of mesenteric lymph nodes and portal and peripheral blood when available were collected under sterile conditions before rat death and cultured in MacConkey agar (Oxoid), Columbia sheep blood (Oxoid), and Esculin-Bile-Azide agar (Merck), and incubated at 37°C for 48 h. Bacterial translocation was defined as a positive culture of mesenteric lymph nodes. Systemic infections were defined as a positive culture of any of the remaining biological samples.

### Statistical analysis

Data are expressed as the mean ± SD statiscal analysis was performed with non-parametric Mann-Whitney test. Standard calculations were performed using SPSS version 16.0. Results were considered statistically significant at *P *< 0.05.

## Results

Rat weight significantly increased in the control group (221 ± 10 g to 355 ± 16 g), remained similar in the group with fatty liver disease (222 ± 12 g to 226 ± 14 g), and decreased in the resveratrol group (218 ± 9 g to 201 ± 11 g). Hepatic index was 4.47 ± 0.63, 4.38 ± 0.34, and 3.9 ± 0.31 in the steatosis, resveratrol, and control groups respectively.

### Histological evaluation

**Figure 2:**
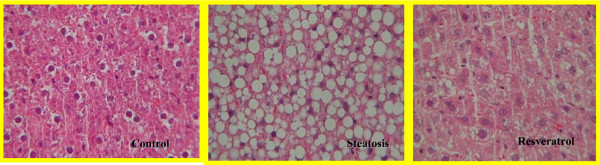
**Histological study in the different groups (control, steatosis, and resveratrol).** Thus, the control group is shown to have no fat vacuoles. A great number of vacuoles were seen in the steatosis group, while the resveratrol group had much less vacuoles of a lower size. Hematoxylin and eosin staining of liver tissue × 40.

No fatty infiltration was seen in the control group (Figure 2). Mean fatty infiltration in the steatosis group was 3 (Table 1). Fat deposit in the steatosis group was classified as macrovesicular. Mean fatty infiltration in the resveratrol group was 1, and fat deposit was mixed. Fatty infiltration in the resveratrol group was significantly lower than in the steatosis group (*P *< 0.05). Inter-observer agreement was 0.84 and intra-observer agreement was 0.79.

**Table 1:** Grades of fatty infiltration in rats and groups.

Group	Rats (n°)	Steatosis grades			
		
		0	1	2	3
Control	12	12	-	-	-
Steatosis	12	-	-	2	10
Resveratrol	12	-	10	2	-

### Laboratory findings

ALT levels were 40 ± 11 IU/L in the control group, 34 ± 9 IU/L in the steatosis group, and 34 ± 11 IU/L in the resveratrol group (*P *< 0.05 between control group vs steatosis and resveratrol groups). Glucose levels were 230 ± 45 mg/dl in the control group, 162 ± 25 mg/dl in the steatosis group, and 145 ± 33 mg/dl in the resveratrol group. Statistically significant differences in glucose levels were seen between the control and steatosis groups and the resveratrol group (*P *< 0.05 between control group vs steatosis and resveratrol groups). Albumin levels were 38.1 ± 2.3 g/dl in the control group, 40.54 ± 1.87 g/dl in the steatosis group, and 41.1 ± 2.9 g/dl in the resveratrol group (*P *< 0.05 between control group vs steatosis and resveratrol groups).

### TNF-α and lipid peroxidation (MDA)

**Figure 3:**
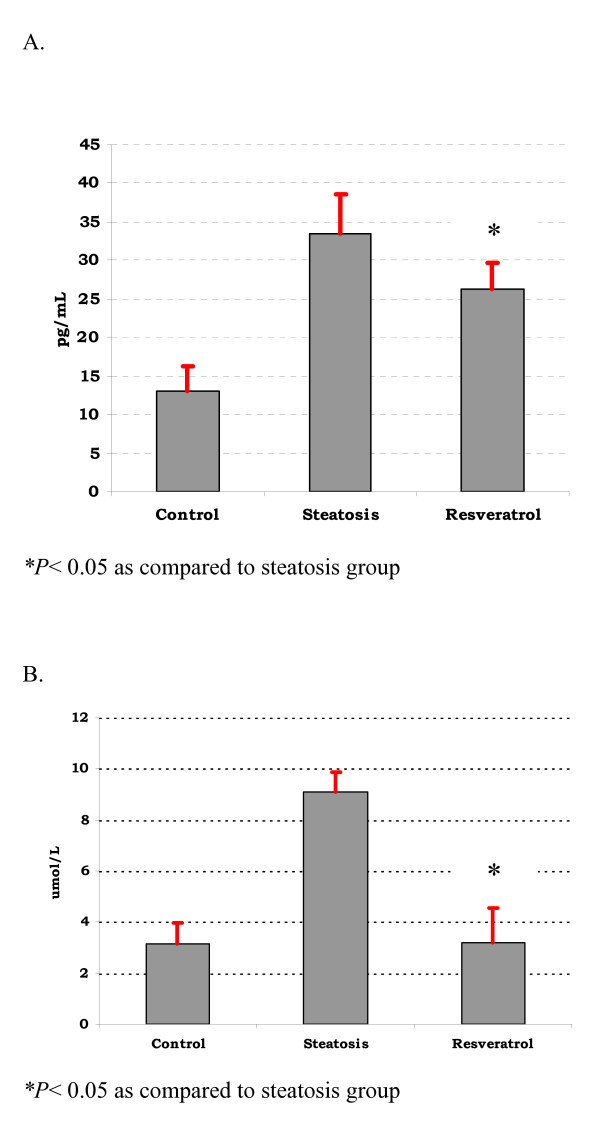
**TNF-α and MDA levels.** A. TNF-α levels in differents groups (pg/mL). TNF-α levels were lower in the resveratrol groups as compared to the control and steatosis groups. B. Levels of MDA in differents groups (μM). MDA levels were lower in the resveratrol groups as compared to the steatosis group (*P < 0.05).

Figure 3 shows that TNF-α levels were increased in the steatosis group as compared to the other two groups (*P *< 0.05). MDA was significantly elevated in the steatosis group as compared to the control group (μM). MDA liver levels were lower in the resveratrol group than in the steatosis group (*P *< 0,001).

### Oxidative stress

The data in table 2 demostrate that resveratrol administration significantly decreased the effects of the stress oxidative in the hepatic tissue.

**Table 2:** Changes of superoxide dismutase, catalase, glutation peroxidasa and nitric oxide synthase in each group.

	**Control**	**Steatosis**	**Resveratrol**
Superoxide dismutase (U/ml)	2.74 ± 2.08	1.15 ± 2.1	3.1 ± 3.2*
Catalase (nmol/min/ml)	3807 ± 315	284 ± 26	713 ± 50*
Glutathione peroxidase (nmol/min/ml)	449 ± 27	6.5 ± 3	406 ± 25*
Nitric oxide synthase (uM)	1.9 ± 0.6	9.17 ± 0.9	4.19 ± 0.7*

*P < 0.05 vs steatosis group

### Bacterial translocation and systemic infections

Cultures of samples of portal blood, peripheral blood, and perihepatic lymph nodes were negative in all three groups tested.

## Discussion

NAFLD represents a wide spectrum of disorders, the hallmark of which is hepatic steatosis. NAFLD was considered a benign condition, but is now increasingly recognized as a major cause of liver-related morbidity and mortality. Insulin resistance is the basis for accumulation of free fatty acids and triglyceride storage in hepatocytes or steatosis. Oxidative stress from steatotic hepatocytes leads to lipid peroxidation, impaired mitochondrial and peroxisomal oxidation of fatty acids, and cytokine release [11]. Endotoxins and endotoxin-inducible cytokines, particularly TNF-α, are required for the pathogenesis of NAFLD in experimental animals. Therefore the TNF-α plays an important role in NAFLD [12,13].

In our study, hepatic steatosis was significantly decreased in rats treated with resveratrol. This effect was associated to a decreased TNF-α production. Different studies have shown that resveratrol decreases TNF-α production. We therefore think that the decreased liver damage in a model of liver steatosis could be related to its anti-TNF-α effect. Other studies [14-16] have shown a relationship between NAFLD and TNF-α levels. Our study shows that TNF-α levels were increased in both the steatosis and resveratrol groups as compared to the control group, suggesting that TNF-α is an important factor in liver damage occurring in NAFLD. However, TNF-α levels were lower in the resveratrol group than in the steatosis group. We therefore think that TNF-α is an important factor for development of this condition. In agreement with other authors [17-19], we did not consider administration of anti-TNF-α or use of another group given repeated TNF-α doses because adequate evidence was already available. Infliximab (anti-TNF-α) reverses the steatosis and the expression of the proinflammatory markers (TNF-α, IL6, IL-1B) and improves insulin signal trasduction in a model of steatosis in rats [20]. The presence of a control group (reference group) allows to compare the effects of the modified diet (high carbohydrate-fat free) on the liver (steatosis group) and the effect of the resveratrol (resveratrol group).

Bacterial translocation from the intestinal lumen to mesenteric lymph nodes is considered to be one of the main events in the pathogenesis of spontaneous bacterial peritonitis and other infections in cirrhosis. TNF-α is involved in the occurrence of bacterial translocation in rats with cirrhosis [21]. We therefore considered whether bacterial translocation acting as a stimulus for TNF-α production occurred in our model. Cultures of samples of portal blood, peripheral blood, and perihepatic lymph nodes were negative in all three groups tested, which rules out this mechanism as responsible for TNF-α elevation.

There are many models of NAFLD liver injuries in animals [22,23]. In rats, cycles of feeding and fasting with hypertonic high calorie diets have been seen to induce fatty liver [8]. In this study, we used feeding and fasting cycles with a modified diet because it is a fast, easy procedure that results in pathological changes similar to those occurring in humans. In our model the deposit of fatty acids and triglyceride storage in hepatocytes is produced by insulin resistance [8,11]. Insulin resistance is the basis for accumulation of free fatty acids and triglyceride storage in hepatocytes, and represents the "first hit" in the pathogenesis of NAFLD [11]. Body mass significantly decreased with the high carbohydrate-fat free diet and dietary restriction. This may be due to a metabolic imbalance of carbohydrate, protein, and fat. However, hepatic index was higher in the group with steatosis as compared to the resveratrol and control groups. Such higher hepatic index occurred despite the increased rat weight in the steatosis group, which means that resveratrol acts by decreasing fat accumulation in the liver and fat weight, and therefore decreases hepatic index.

ALT is a relatively liver-specific aminotransferase. Elevation of ALT activity in serum is the result of leakage from damaged cells and therefore reflects hepatocyte damage. Elevated transaminase levels correlated strongly with NAFLD [2]. ALT levels were significantly lower in the resveratrol group as compared to the control group (34 IU/L versus 40 IU/L) and similar to steatosis group levels. In the group treated with resveratrol, lower glucose levels and higher serum albumin levels as compared to untreated rats were also found. Studies have reported increases in serum TNF-α levels in humans with insulin resistance [24]. Hyperglycemia and insulin resistance are associated to the presence of NAFLD [2,25]. Changes in plasma levels of markers predicting for the onset of diabetes occurred with a high carbohydrate-fat free diet [25]. Glucose levels were decreased in the resveratrol group [26], as occurred in our study. Other studies have also noted that resveratrol improved insulin sensitivity, lowered plasma glucose, and increased mitochondrial capacity in obese mice [27]. Insulin resistance was not analyzed in our study.

High hepatic MDA levels were found in the steatosis group, in agreement with other studies [28]. Resveratrol improved MDA levels. Oxidative stress is believed to play an important role in pathogenesis of NAFLD. It is likely to be involved in disease progression from steatosis to steatohepatitis and potentially cirrhosis. It has been shown that chronic oxidative stress, generated through oxidation of cytotoxic free fatty acids, may lead to cytokine upregulation and depletion of hepatic antioxidant levels [29,30]. In addition, enhanced lipid peroxidation leads to the generation of by-products, such as MDA, which have been shown to further stimulate cytokine production. They are involved in hepatic stellate cell activation, fibrogenesis, and enhanced extracellular matrix protein deposition [28]. Resveratrol caused increased of hepatic antioxidant levels as superoxide dismutase, glutathione peroxidase and catalase and decreased nitric oxide synthase in the liver.

Fatty acid oxidation is an important source of reactive oxygen species in fatty livers. Some consequences of increased reactive oxygen species levels include an impaired protein stability, membrane destruction via lipid peroxidation, and release of proinflammatory cytokines (increased TNF-α levels) [31]. Reactive oxygen species may attack polyunsatured fatty acids and initiate lipid peroxidation within the cell, which results in MDA formation. Oxidants may not only act as toxic substances, but also as second messengers (activation of transcription factor NF-kB) [32]. Resveratrol increases insulin sensitivity and insulin-like growth factor-1 levels (IGF-1) [28]. Resveratrol would also act by decreasing lipid peroxidation and reactive oxygen species release, thereby decreasing inflammatory response and liver lesions [33,34]

Recently, researches suggest that other mechanisms the resveratrol improve in NAFLD are by the activation of AMP-activated protein kinase and the activation of SIRT1 [35-37].

## Conclusion

In summary, our study shows that resveratrol decreases liver steatosis in rats and that its effect is mediated, at least partly, by TNF-α and antioxidant activities. In our model, bacterial translocation was not responsible for TNF-α elevation. Further studies are warranted to determine whether resveratrol decreases or prevents liver steatosis.

## Competing interests

The authors declare that they have no competing interests.

## Authors' contributions

LB, EH, MB, PA, and NG–U participated in study conception and design, manuscript preparation, and practical conduct of the study. CS collected and analyzed data and was involved in study conduct. AC and JA critically reviewed the manuscript. AG and BI carried out biochemical and microbiological tests. ML and EH performed the pathological examinations. All authors have read and approved the manuscript.

## Pre-publication history

The pre-publication history for this paper can be accessed here:


